# Structural Determinants of Antiretroviral Therapy Use, HIV Care Attendance, and Viral Suppression among Adolescents and Young Adults Living with HIV

**DOI:** 10.1371/journal.pone.0151106

**Published:** 2016-04-01

**Authors:** Shoshana Y. Kahana, Richard A. Jenkins, Douglas Bruce, Maria I. Fernandez, Lisa B. Hightow-Weidman, Jose A. Bauermeister, Adolescent Medicine Trials Network for HIV/AIDS Interventions

**Affiliations:** 1 Division of Epidemiology, Services, and Prevention Research, National Institute on Drug Abuse/National Institutes of Health, Bethesda, MD, United States of America; 2 Department of Health Sciences, DePaul University, Chicago, IL, United States of America; 3 Department of Preventive Medicine and Department of Public Health Program, College of Osteopathic Medicine, Nova Southeastern University, Ft. Lauderdale, FL, United States of America; 4 Department of Medicine, Division of Infectious Diseases, University of North Carolina at Chapel Hill, Chapel Hill, NC, United States of America; 5 Department of Health Behavior and Health Education, School of Public Health, University of Michigan, Ann Arbor, MI, United States of America; British Columbia Centre for Excellence in HIV/AIDS, CANADA

## Abstract

**Background:**

The authors examined associations between structural characteristics and HIV disease management among a geographically diverse sample of behaviorally and perinatally HIV-infected adolescents and young adults in the United States.

**Methods:**

The sample included 1891 adolescents and young adults living with HIV (27.8% perinatally infected; 72.2% behaviorally infected) who were linked to care through 20 Adolescent Medicine Trials Network for HIV/AIDS Interventions Units. All completed audio computer–assisted self-interview surveys. Chart abstraction or blood draw provided viral load data. Geographic-level variables were extracted from the United States Census Bureau (e.g., socioeconomic disadvantage, percent of Black and Latino households, percent rural) and Esri Crime (e.g., global crime index) databases as Zip Code Tabulation Areas. AIDSVu data (e.g., prevalence of HIV among youth) were extracted at the county-level. Using HLM v.7, the authors conducted means-as-outcomes random effects multi-level models to examine the association between structural-level and individual-level factors and (1) being on antiretroviral therapy (ART) currently; (2) being on ART for at least 6 months; (3) missed HIV care appointments (not having missed any vs. having missed one or more appointments) over the past 12 months; and (4) viral suppression (defined by the corresponding assay cutoff for the lower limit of viral load at each participating site which denoted nondetectability vs. detectability).

**Results:**

Frequencies for the 4 primary outcomes were as follows: current ART use (n = 1120, 59.23%); ART use for ≥6 months (n = 861, 45.53%); at least one missed HIV care appointment (n = 936, 49.50); and viral suppression (n = 577, 30.51%). After adjusting for individual-level factors, youth living in more disadvantaged areas (defined by a composite score derived from 2010 Census indicators including percent poverty, percent receiving public assistance, percent of female, single-headed households, percent unemployment, and percent of people with less than a high school degree) were less likely to report current ART use (OR: 0.85, 95% CI: 0.72–1.00, p = .05). Among current ART users, living in more disadvantaged areas was associated with greater likelihood of having used ART for ≥6 months. Participants living in counties with greater HIV prevalence among 13–24 year olds were more likely to report current ART use (OR: 1.32, 95% CI: 1.05–1.65, p = .02), ≥6 months ART use (OR: 1.32, 95% CI: 1.05–1.65, p = .02), and to be virally suppressed (OR: 1.50, 95% CI: 1.20–1.87, p = .001); however, youth in these areas were also more likely to report missed medical appointments (OR: 1.32, 95% CI: 1.07–1.63, p = .008).

**Conclusions:**

The findings underscore the multi-level and structural factors associated with ART use, missed HIV care appointments, and viral suppression for adolescents and young adults in the United States. Consideration of these factors is strongly recommended in future intervention, clinical practice, and policy research that seek to understand the contextual influences on individuals’ health behaviors.

## Background

Human immunodeficiency virus (HIV) incidence rates are high and continue to increase among adolescents and young adults in the United States (US), particularly for young men who have sex with men (MSM) and transgender populations [1, 2]. In addition to troublingly high rates of HIV acquisition, youth and young adults living with HIV struggle with obtaining access to and adhering to antiretroviral therapy (ART), resulting in low rates of viral suppression [3, 4]. Poor ART adherence among youth has been attributed to individual-level factors, including drug use, mental health conditions (depression, trauma), and limited social support [5–9]. Factors related to HIV illness (e.g., behavioral vs. perinatal HIV acquisition, length of time since diagnosis) and care (e.g., access to quality care, provider-patient relationship and practices) are also related to adherence and viral suppression among adolescents and young adults living with HIV [4, 10–11].

Increasingly, efforts aiming to reduce the gaps across the HIV continuum of care have highlighted the need to identify and address the individual and structural factors creating these disparities. In prior analyses, we identified individual-level correlates of ART use, missed appointments, and viral suppression among a sample of perinatally and behaviorally HIV-infected adolescents and young adults in the US [4]. For perinatally infected adolescents and young adults, consistent HIV care and lack of current substance abuse were significant correlates of ART use; these variables and non—African American race were among the factors associated with virologic suppression for PIY. For behaviorally infected adolescents and young adults, heterosexual orientation, employment, and higher levels of education were significantly related to ART use, while viral suppression was related to ART use and consistent attendance at HIV care.

There is increasing recognition that structural factors (defined broadly as barriers to or facilitators that may relate to economic, social, policy, systems, organizational or other aspects of the environment) play a key role in heightening youths’ vulnerability to HIV-related correlates and outcomes, such as acquisition and transmission behaviors. Individuals living in more structurally disadvantaged settings, including living in poverty, chronic unemployment, high rates of school dropout, racial/ethnic segregation, and in locations with high HIV prevalence rates are more likely to both encounter and manifest high risk health risk factors than counterparts living in more advantaged neighborhoods [12], see review article [13]. In a seminal review article, Latkin [13] reviews various mechanisms through which factors in an individual’s environment can affect health risk behavior, including: a) risk environment and behavioral setting, which has been defined as space in which a variety of factors interact to increase the chances of harm or maladaptive behaviors occurring; and/or b) neighborhood disorder in which impoverished neighborhoods suffer from physical signs of decay (e.g., abandoned buildings, litter, and graffiti) and are linked to concentrated health consequences through social disorganization (lack of social control mechanisms). At their core, many theories posit that environments with structural disadvantages are negatively impacted through decreased collective efficacy and social cohesion, which in turn many affect the individual’s ability to engage in health seeking behaviors (e.g., engage in or be maintained in HIV care).

Many studies have focused on how broader structural factors are related to HIV risk behaviors, acquisition, incidence, and prevalence [14–22]. Fewer articles have been published on these factors as they relate to the continuum of HIV care, and specifically use of ART as well as engagement and retention in HIV care. This is particularly important as delineating structural factors can often be valuable in characterizing the HIV epidemic and treatment cascade within and across various, often heterogeneous, geographies and communities, and thus informing the development of specific individual-level or structural-levels interventions that may be needed. Shacham et al. [23] for example, found that individuals residing in neighborhoods with higher poverty rates were more likely to have lower CD4 cell counts, while those in neighborhoods with higher rates of unemployment were less likely to have a current antiretroviral prescription. The availability of transportation to appointments and health insurance has also been associated with successful linkage and retention in care for HIV-infected adolescents [24]. Finally, Eberhart [25] reported that in specific geographic areas in Philadelphia, factors significantly associated with residence in poor viral suppression hotspots included higher economic deprivation and shorter distance to pharmacies.

In this paper, the authors examine the association between structural-level characteristics and HIV disease management among a large, geographically diverse sample of HIV-infected youth. Specifically, we explore the interplay of structural-level and individual-level factors as they relate to antiretroviral therapy use, consistent appointment keeping, and viral suppression among adolescents and young adults living with behaviorally and perinatally acquired HIV in the US. We hypothesize that structural-level factors, including socioeconomic status (SES), exposure to crime, burden of HIV (combined incidence and prevalence) among young adults (13–24 year olds) will be associated with lower rates of ART use (current and for at least 6 months), greater number of missed HIV care appointments and lower rates of viral suppression.

## Methods

The sample frame and procedures for the current study have been described elsewhere [4]. In brief, from December 2009 to June 2012, a total of 2,225 adolescents and young adults linked to care at units or clinics associated with the Adolescent Medicine Trials Network for HIV/AIDS Interventions (ATN) were recruited to participate in a cross-sectional survey. The 20 clinics were broadly distributed geographically within the continental US and Puerto Rico and are situated in cities where the HIV prevalence and incidence rates are among the highest in the US (see Acknowledgments for cities represented as well as map in S1 Fig.

To be eligible, youth had to be (1) between 12 and 26 years of age (inclusive), (2) HIV infected, (3) aware they were HIV infected, (4) linked to or receiving care in one of the ATN’s clinical sites or affiliates (e.g., had at least 1 clinic visit during the enrollment period), and (5) able to understand English or Spanish. The study was approved by the Institutional Review Boards (IRB) at each participating site and those from the members of the protocol team. Research staff members approached all youth meeting eligibility criteria during one of their scheduled clinic visits. After a thorough explanation of the study, staff members obtained signed informed consent or assent from youth agreeing to participate. Waiver of parental consent was done on a site by site basis. Although the majority of IRBs granted a waiver of parental consent, the team obtained written parental permission when required. Within 2 weeks of providing consent, participants completed audio computer—assisted self-interviews to assess psychosocial and health factors, which took approximately 45–90 minutes. Participants were given a small incentive determined by the sites’ IRB as compensation for their time.

### Individual-Level Data Measures

#### ART use & adherence

Using findings from previous research [26], ATN scientists developed a 25-item questionnaire to assess medication regimen, frequency of dosing and number and type of pills prescribed per day, and number of doses missed in the last 7 days. Participants who self-reported being on ART and had a current regimen identified during medical chart review were classified as “currently on ART.” Given that 6 months on an effective ART regimen should be sufficient to result in viral suppression [27–28], we also included “on ART for at least 6 months” as a variable of interest in our analyses.

#### Missed HIV care appointments

Adherence to scheduled medical appointments with HIV care provider over the past 12 months was assessed by self-reported number of missed visits. This measure has been one of the most widely used retention measures in the literature and has been applied as both a dichotomous and as a count measure [29–31]. We dichotomized the missed appointments variables as not having missed any medical appointments or having missed one or more appointments. Given prior data suggesting that youth are more prone to experience difficulties in adhering to their medical visits, we created an additional dichotomous variable comparing youth who had missed ≤1 visit to youth who reported more than one missed visit, regardless of how many visits were scheduled. The results did not change substantively when we used either outcome; thus, for comparison to the larger literature, the more conservative indicator (none vs. any) was reported in this manuscript.

#### Viral load

Viral suppression rates were only calculated for those youth who were on ART for at least 6 months. Plasma HIV-1 RNA level (VL) and CD4+ count data obtained within the prior 6 months were abstracted from medical records. The minority (approximately 5%) of participants who did not have VL and CD4 evaluations within 6 months of the study had blood collected at the baseline visit for these measurements. Because of the variability in type of VL assay used across the study sites (i.e., Bayer/Siemens Versant HIV-1 RNA 3.0 (bDNA), Roche Amplicor HIV-1 Monitor—Standard/Ultrasensitive, Roche COBAS AmpliPrep/COBAS TaqMan HIV-1 Test, v1.0, 2.0, and Abbott RealTime HIV-1 Assay), the corresponding assay cutoff for the lower limit of VL (LLD) was used. A dichotomous variable to designate virally suppressed (nondetectable) or virally nonsuppressed (detectable) was created.

#### Demographics

Participants reported demographic information, including age, sex assigned at birth and current self-identified gender identity. The authors created four gender identity groups: cisgender females (i.e., females whose identified gender and sex assigned at birth accord), cisgender males (i.e., males whose identified gender and assigned sex at birth accord), transgender males (i.e., males who were assigned a female sex at birth), and transgender females (i.e., females who were assigned a male sex a birth). We also ascertained participants’ race and ethnicity, sexual orientation identity, educational attainment, and employment status. HIV acquisition route (behavioral or perinatal) and time since diagnosis were collected as well.

#### Mental health

Mental health symptoms were assessed with the Brief Symptom Inventory [32], which yields 9 primary symptom scales and a global severity index (GSI) with gender and aged-specific norms. The GSI reflects an overall evaluation of a respondent’s psychological distress level. Clinically indicative scores were age and gender normed: GSI ≥ 1.71 for males ≤19 years old; GSI ≥ 1.59 for females ≤ 19; GSI ≥ 0.58 for males ≥ 20; and GSI ≥ 0.78 for females ≥ 20.

#### Substance use

The Alcohol, Smoking and Substance Involvement Screening Test (ASSIST) [33] was used to assess substance use behaviors. The ASSIST is an 8-item questionnaire that assesses the frequency and consequences of substance use. For current use (past 3 months), any response of 4 (indicating daily or almost daily use) for any substance was considered “problematic substance use.”

#### Sexual behavior and activity

Using findings from previous research, ATN scientists developed a 38-item questionnaire to assess sexual activity. Participants reported the number of sex partners and the frequency of condom-protected and condomless oral, vaginal, and anal sexual activity with HIV-infected and HIV-negative/unknown status female and male partners during the past 3 months.

### Structural-level data measures

Geographically dependent variables were collected from a number of existing sources (described below) and appended to the data collected at clinical sites using participant residential zip code as a geographical identifier. To align census-derived data with participant residential zip codes we used the 2010 Zip Code Tabulation Area (ZCTA), a spatial unit developed by the U.S. Census Bureau to be comparable to zip codes (http://www.census/gov/geo/reference/zctas.html). For HIV-related data (i.e., AIDSVu), we used county-level aggregate indicators, as many health departments do not disclose the number of diagnosed HIV cases by zipcode to circumvent unintended disclosure.

#### Racial/ethnic composition

In order to examine the racial/ethnic composition of the ZCTAs representing the ATN sample, we extracted data from the 2010 Census regarding the percent of African American/ Black and percent of Latino/Hispanic individuals living within each ZCTA.

#### Urban/rural

Using data from the 2010 Census, we estimated the percent of individuals living in an area designated as urban/rural for each ZTCA. Given that the majority of ZTCAs were predominately urban (reflecting to a large extent the urban context where ATN sites are located), we created a dichotomous variable that indicated whether a ZTCA had at least 5% of its residents located within a rural setting.

#### Socioeconomic disadvantage

For each ZCTA, we created a standardized concentrated economic disadvantage score based on the following 2010 Census indicators: percent poverty, percent receiving public assistance, percent of female, single-headed households, percent unemployment, and percent of people with less than a high school degree. We created the standardized disadvantaged score using Principal Axis factor analysis with Varimax rotation. Consistent with prior studies [17, 22, 34], this composite score (α = 0.87) had a one-factor solution that explained 58.27% of the variance. Higher scores indicate greater socioeconomic disadvantage.

#### Crime rate

We created a standardized score based on the total crime index from the 2010 Esri Crime database. The total crime index is typically comprised of the following offenses: willful homicide, forcible rape, robbery, burglary, aggravated assault, larceny over $50, motor vehicle theft, and arson. Per the USA Crime Index, “the crime index compares the average local crime level to that of the United States as a whole. An index of 100 is average. A crime index of 120 indicates that crime in that area is 20 percent above the national average.” We standardized this index to a z-score. Our standardized mean crime index was -.04, suggesting that youth in our study lived in areas with slightly lower crime rates as compared to the national average.

#### HIV prevalence

HIV prevalence data were obtained for the counties represented in our study (counties were linked to individual participants through ZCTAs) using the AIDSVu database. Given the nature of these data, HIV prevalence data cannot be linked to ZCTAs or zipcodes if it may result in identifiable data. Given our interest in HIV burden among youth, we then computed the percent of all HIV cases in a county accounted by youth ages 13 to 24 (inclusive). Given the skew of this variable and recognizing that the age distribution may vary across ZCTAs, we estimated the median percentage (13%) and dichotomized the HIV prevalence indicator using this estimate (i.e., 0 = diagnosed HIV cases among youth are less than or equal to 13% of the total prevalence in the county; 1 = diagnosed HIV cases among youth account for more than 13% of the total prevalence in the county). We included HIV prevalence as a structural-level correlate, consistent with other research that suggests that HIV prevalence is often used to locate service sites [35].

### Data analysis

The final sample included 1891 individuals and 733 distinct ZCTAs. Prior to analysis, we deleted 257 participants with missing zip code information and an additional 20 participants linked to 12 invalid zipcodes. An additional 57 participants were excluded because they had one or more of the following: missing VL data, reported VLs that did not correspond with the LLD of the reported assay at their site, or with unclear acquisition mode of HIV-infection.

Descriptive analyses were conducted using PASW (SPSS), version 18.0. We computed frequencies, means and other measures of central tendency for individual- and structural- level factors described above. Multi-level models were then developed using HLM software (version 7; SSI, Inc.) to examine individual- and structural- level factors associated with (1) current ART use; (2) being on ART for (at least) ≥6 months; (3) missed HIV care appointments; and (4) viral suppression. For these models, a Bernoulli distribution was employed to account for the dichotomous nature of our outcomes.

We used a means-as-intercept model [36] to examine the association between our outcomes of interest and our individual- and structural-level variables, partitioning each outcome’s variance by its individual- (Level One) and structural-level (Level Two) components concurrently to account for the nested structure of our data. Within the means-as-intercept model, we allow the mean score (i.e., model intercept) to vary at random. We followed a two step-modeling approach. In the first model, we entered our structural-level variables after ensuring that our structural indicators were not highly correlated (*r* > .65) with each other. This model step allowed us to test whether the random variation could be explained by our structural-level data. Structural-level variables of interest included crime index (z-score), socioeconomic disadvantage (z-score), percentage racial and ethnic minority (African American and Hispanic) in any given ZCTA, percentage of counties in which HIV prevalence among 13 to 24 year olds was above the median HIV prevalence (13%), and the percentage of ZCTAs where five percent or more of residents were identified as living in a rural area.

In the second model for each outcome, we proceeded to enter the individual-level factors identified in our prior ATN publication as being associated with HIV continuum of care outcomes. We followed the same strategy for each outcome of interest; however, for our viral suppression analyses, we had difficulty stabilizing our models due to limited cell observations across our transgender participants. Consequently, we merged transgender men and transgender women into a single indicator (e.g., transgender identity) for this analysis. We treated the slopes between covariates and outcomes as fixed effects, as we did not have a priori hypotheses suggesting random effects in these associations [37].

## Results

### Participant characteristics

Of the 1891 participants in the analysis sample, the majority were African American (n = 1314, 69.49%), identified as male (n = 1205, 63.72%) and behaviorally HIV-infected (n = 1366, 72.24%). Mean age of the sample was 20.32 years (SD = 2.67) and over half (n = 1046, n = 55.32%) of the sample identified as a sexual minority (gay, bisexual). Approximately 5 individuals (or less than 1% of the sample) had been infected via injection drug use as compared to 1192 youth and young adults (87.26% of the sample) who identified sex with a man as the mode of transmission. Thirty percent (n = 568) had been diagnosed with HIV in the past year. Over half of the sample reported current ART use (n = 1120, 59.23%) and less than half the sample (n = 861, 45.53%) reported ART use for at least 6 months or missing at least one HIV care appointment over the past year (n = 936, 49.50%). Just less than a third were virally suppressed (n = 577, 30.51%). Finally, a significant number reported problematic substance use (n = 658, 34.80%) and exhibited elevated psychological distress (n = 819, 47.12%). Participant characteristics of the total sample appear in Table 1.

**Table 1:** Demographic, Biomedical, and Psychosocial/Behavioral Characteristics of Perinatally and Behaviorally HIV-Infected Adolescents and Young Adults Linked to Care (n = 1891).

Variable	No. (%) or *Mean* ± (SD)
Age	20.32 (2.66)
Self-Identified Gender	
Males (cismale)	1205 (63.72)
Females (cisfemale)	47 (2.49)
Transgender	633 (33.47)
No answer/Missing	6 (.03)
Race	
African American	1314 (69.49)
White	221 (11.69)
Mixed Race	191 (10.10)
Asian/Pacific Islander	16 (.85)
Native American	15 (.79)
Other	119 (6.29)
No answer/missing data	15 (.79)
Ethnicity	
Latino/Hispanic	363 (19.20)
Sexual Orientation	
Heterosexual	842 (44.53)
Gay/Lesbian	746 (39.45)
Bisexual	245 (12.96)
Questioning/Queer/Other	55 (2.91)
No answer/missing data	3 (.16)
Highest Level of Education	
No High School Completion	625 (33.05)
High School Graduate/GED	656 (34.69)
Some College/College or Technical School Graduate	594 (31.41)
No answer (missing data)	16 (.85)
Currently Employed (Full or Part Time)	644 (34.01)
Stably Housed	1800 (95.19)
Route of Acquisition	
Perinatal	525 (27.76)
Behavioral	1366 (72.24)
Length of Time since HIV Diagnosis (Days)	
0–12 mos.	568 (30.03)
13–24 mos.	290 (15.34)
25+ mos.	1033 (54.63)
VL	
Suppressed (Undetectable)	577 (30.51)
Nonsuppressed (Detectable)	1314 (69.49)
Current ART Use	1120 (59.2)
ART Use for ≥6 months	861 (45.53)
Adherence to ART (last 7 days)	86.67 (22.93)
≥90 Adherence (among those on ART)	691 (61.70)
Missed # of HIV care appointments over past 12 months	
None	955 (50.50)
One or more	936 (49.50)
Sex Risk (past 3 months)	
Any unprotected sex	592 (31.31)
Any unprotected sex with serodiscordant/status unknown partner	433 (22.90)
Total number of partners (≥2)	813 (42.99)
ASSIST Current (≥4 on any substance)	658 (34.80)
GSI, Clinically indicative scores	819 (43.31)

Notes. VL, viral load; ART, antiretroviral therapy; ASSIST, the Alcohol, Smoking and Substance Involvement Screening Test; GSI, Global Severity Index on the Brief Symptom Inventory. Results of continuous measures are *italicized*.

### Structural Characteristics

Structural-level characteristics are described in Table 2. In general, there were fairly high rates of crime exposure in the ZCTAs as well as high rates of SES disadvantage, HIV prevalence rates, and urbanity (which is expected given how ATN sites were originally selected into the project).

**Table 2:** Description of ZCTA- and County-Level Derived Structural-Level Characteristics.

Variable	Level	N	Mean	SD	Min	Max
Average Population Size	ZCTA	785	36723.24	20254.57	204	113916
Total Crime Index (z-scored)	ZCTA	733	-0.37	0.91	-1.67	3.25
Socioeconomic Disadvantage	ZCTA	733	-0.45	0.92	-2.71	2.86
% Below Poverty		733	0.20	0.12	0.00	1.00
% Unemployed		733	0.07	0.03	0.02	0.20
% Getting Public Aid		733	0.04	0.03	0.00	0.23
% High School Degree		733	0.81	0.09	0.54	1.00
% Single-mother Household		733	0.19	0.09	0.00	0.46
Percent Black	ZCTA	733	0.33	0.29	0.00	0.98
Percent Hispanic	ZCTA	733	0.23	0.23	0.00	0.98
AIDSVU County Rate*	County	733	773.89	590.36	80.00	2451.00
Percent of HIV Cases, 13–24 year olds	County	733	0.24	0.07	0.06	0.64
Percent of Areas with Youth HIV Prevalence above 13%	County	733	0.40	0.49	0.00	1.00
Percent of Areas where at least 5% of residents live in rural classification	ZCTA	733	.09	0.29	0.00	1.00

* = County Rate of adults and adolescents living with an HIV or AIDS diagnosis per 100,000 population.

Note: ZCTA, ZIP Code Tabulation Areas; SD, Standard deviation; Min, minimum, Max, maximum.

### Multi-level models

#### Current ART use

Youths’ likelihood of current ART use was associated with their counties’ socioeconomic disadvantage score (OR: .80, 95% CI: 0.69–0.94, p = .005), with youth living in more socioeconomically-disadvantaged areas being less likely to report current ART use. We found no additional structural-level associations in this model (see Table 3).

**Table 3:** Structural-Level Correlates of Current ART Use, ≥6 Months ART Use, and Missed HIV Care Appointments.

	Current ART Use			ART ≥6 Months Use			Missed Appointments		
	OR	95% CI	*p*	OR	95% CI	*p*	OR	95% CI	*p*
Mean Score, *β*_*0*_	1.41	(1.22,1.63)	.001	5.60	(2.20,5.88)	.001	0.98	(0.72,1.33)	.88
Crime Index Score, *γ*_*01*_	1.03	(0.91,1.17)	.66	0.94	(0.79,1.12)	.49	1.00	(0.90,1.11)	.99
Socioeconomic Disadvantage, *γ*_*02*_	0.80	(0.69,0.94)	.005	1.21	(0.96,1.53)	.11	1.23	(1.06,1.43)	.007
% Black, *γ*_*03*_	1.18	(0.68,2.07)	.56	0.70	(0.32,1.56)	.38	0.77	(0.46,1.31)	.34
% Hispanic, *γ*_*04*_	1.39	(0.75,2.60)	.30	0.67	(0.29,1.56)	.36	0.82	(0.46,1.44)	.49
Youth HIV Prevalence >13%, *γ*_*05*_	1.20	(0.97,1.49)	.10	1.25	(0.94,1.66)	.13	1.23	(1.01,1.49)	.04
% Rural > 5%, *γ*_*06*_	1.25	(0.77,2.02)	.37	1.54	(0.67,3.45)	.30	0.87	(0.54, 1.40)	.56

Notes. OR = Odds Ratio; CI = Confidence Interval; ART = Antiretroviral therapy

Once we entered structural-level factors into the model (see Table 4), we found that adolescents and young adults living in areas with more economic disadvantage remained less likely to be on ART (OR: .85, 95% CI: 0.72–1.00, p = .05). Youth living in counties with greater HIV prevalence among 13–24 year olds were more likely to report current ART use (OR: 1.32, 95% CI: 1.05–1.65, p = .02). Among individual-level variables in these multi-level analyses, cisgender females (females whose identified gender and birth sex accord) were less likely to report current ART use than cisgender males (males whose identified gender and birth sex accord; OR: .56, 95% CI: 0.42–0.75, p < .001). In addition, perinatally infected youth were more likely to report current ART use than behaviorally infected counterparts (OR: .40, 95% CI: 0.28–0.57, p < .001). Current ART use was more likely for youth who had completed high school (OR: 1.44, 95% CI: 1.12–1.84, p = .004) and with longer timeframes since HIV diagnosis (OR: 1.24, 95% CI: 1.19–1.29, p < .001). Finally, youth were less likely to report current ART use if they met criteria for problematic substance use (OR: .75, 95% CI: 0.57–0.98, p = .04) or engaged in any recent unprotected vaginal or anal sex (OR .72, 95% CI: 0.57–0.91, p = .007). We observed no significant associations between current ART use and psychological distress, transgender identity, sexual orientation, race/ethnicity, or employment status.

**Table 4:** Multi-level Associations between Current ART Use, ≥6 Month ART Use, and Missed HIV Care Appointments and Structural- and Individual- Level Characteristics.

	Current ART Use			ART ≥6 Months Use			Missed Appointments		
	OR	95% CI	*p*	OR	95% CI	*p*	OR	95% CI	*p*
Mean Score, *β*_*0*_	4.81	(2.97,7.80)	.001	6.27	(2.62,15.01)	.001	0.28	(0.17,0.47)	.001
Crime Index Score, *γ*_*01*_	1.08	(0.94,1.24)	.29	1.06	(0.87,1.29)	.58	1.02	(0.91,1.14)	.79
Socioeconomic Disadvantage, *γ*_*02*_	0.85	(0.72,1.00)	.05	1.32	(1.00,1.75)	.05	1.15	(0.98,1.35)	.08
% Black, *γ*_*03*_	1.15	(0.61,2.19)	.66	0.39	(0.15,1.04)	.06	0.74	(0.42,1.31)	.30
% Hispanic, *γ*_*04*_	1.24	(0.60,2.55)	.56	0.54	(0.20,1.48)	.23	0.86	(0.47,1.58)	.62
% Youth HIV Prevalence >13%, *γ*_*05*_	1.32	(1.05,1.65)	.02	1.60	(1.15,2.24)	.006	1.32	(1.07,1.63)	.008
% Rurality > 5%, *γ*_*06*_	0.96	(0.56,1.66)	.89	1.37	(0.62,3.07)	.44	1.02	(0.64,1.62)	.93
Behaviorally-infected, *β*_*1*_	0.40	(0.28,0.57)	.001	0.52	(0.30,0.90)	.02	1.26	(0.91,1.74)	.17
Cis-Female, *β*_*2*_	0.56	(0.42,0.75)	.001	0.95	(0.61,1.50)	.84	1.10	(0.85,1.42)	.47
Transgender Male, *β*_*3*_	0.29	(0.07,1.25)	.10	0.27	(0.02,4.10)	.35	2.69	(0.63,11.43)	.18
Transgender Female, *β*_*4*_	1.24	(0.67,2.31)	.50	0.75	(0.32,1.79)	.52	1.41	(0.75,2.63)	.29
Aged 18 years of age or older, *β*_*5*_	0.75	(0.51,1.11)	.15	0.67	(0.36,1.25)	.20	1.85	(1.29,2.66)	.001
White Identified, *β*_*6*_	0.88	(0.67,1.16)	.37	1.65	(1.11,2.47)	.01	1.16	(0.89,1.51)	.27
Hispanic Ethnicity, *β*_*7*_	0.98	(0.72,1.34)	.89	1.58	(0.96,2.61)	.07	1.07	(0.80,1.45)	.64
Heterosexual identity, *β*_*8*_	0.96	(0.72,1.28)	.77	1.60	(1.02,2.51)	.04	0.95	(0.73,1.24)	.73
Employed, *β*_*9*_	1.20	(0.96,1.51)	.11	0.96	(0.67,1.38)	.83	0.86	(0.70,1.07)	.17
Completed High School, *β*_*10*_	1.44	(1.12,1.84)	.004	1.01	(0.69,1.50)	.95	0.76	(0.60,0.96)	.02
Mental Health Symptoms, *β*_*11*_	1.03	(0.83,1.28)	.81	0.74	(0.52,1.04)	.08	1.63	(1.32,2.02)	.001
Problematic Substance Use, *β*_*12*_	0.75	(0.57,0.98)	.04	0.85	(0.58,1.25)	.41	1.74	(1.37,2.21)	.001
Recent Unprotected Sex, *β*_*13*_	0.72	(0.57,0.91)	.007	0.79	(0.54,1.17)	.25	1.07	(0.84,1.35)	.59
Time Since Diagnosis, *β*_*14*_	1.24	(1.19,1.29)	.001	1.43	(1.33,1.54)	.001	1.09	(1.04,1.13)	.001

Notes. OR = Odds Ratio; CI = Confidence Interval; ART = Antiretroviral therapy

#### On ART >6 months

We observed no association between structural-level indicators and use of ART for at least 6 months in our structural-only model (see Table 3); however, once we included individual-level characteristics into our analyses (see Table 4), we found that ART use for ≥6 months was more common among youth living in locations with more economic disadvantage (OR: 1.32, 95% CI: 1.00–1.75, p = .05) where youth accounted for a greater HIV prevalence of HIV cases (OR: 1.60, 95% CI: 1.2.24, p = .006). Among individual-level variables, perinatally infected youth were more likely to have been on ART for ≥6 months than behaviorally infected peers (OR: .52, 95% CI: 0.30–0.90, p = .02). White youth were more likely to report ART for ≥6 months as compared to non-White participants (OR: 1.65, 95% CI: 1.11–2.47, p = .01). Heterosexual youth were also more likely to report ART for ≥6 months than sexual minority peers (OR: 1.60, 95% CI: 1.02–2.51, p = .04). Likelihood of ART use for at least 6 months was also greater among youth who had been diagnosed with HIV for a longer period of time (OR: 1.43, 95% CI: 1.33–1.54, p < .001). We found no significant associations between ART use for at least 6 months and age, Latino ethnicity, employment status, education, problematic substance use or psychological distress, condomless oral, vaginal, and anal sexual activity, or gender identity.

#### Missed HIV care appointments

Missed appointments were reported more often by individuals from more economically disadvantaged ZCTAs (OR 1.23, 95% CI 1.06–1.43, p = .007) and from counties with greater HIV prevalence among youth (OR 1.23, 95% CI 1.01–1.49, p = .04; see Table 3). While the relationship between county HIV prevalence among youth and missed appointments persisted once we entered the individual-level variables, we observed that the relationship between ZCTA-derived socioeconomic disadvantage and missed appointments was suppressed (see Table 4). Among individual-level variables, the likelihood of missed appointments was greater among older youth (OR: 1.85, 95% CI: 1.29–2.66, p < .001) and a longer time since HIV diagnosis (OR: 1.09, 95% CI: 1.04–1.13, p < .001). Youth with a high school education or greater (OR: .76, 95% CI: 0.60–0.96, p = .02) were less likely to have missed appointments. Likelihood of missed appointments was greater among youth who met criteria for problematic substance use (OR: 1.74, 95% CI: 1.37–2.21, p < .001) and psychological distress (OR: 1.63, 95% CI: 1.32–2.02, p < .001). There were no significant associations between missed appointments and race, ethnicity, sexual orientation, employment status, unprotected sex, or gender identity.

#### Viral suppression

Individuals who lived in counties with greater HIV prevalence among youth (OR: 1.43, 95% CI: 1.17–1.75, p < .001) were more likely to be virally suppressed (see Table 5). Once we entered individual-level variables into the model, we noted that cis-gender females were less likely to be virally suppressed than cis-gender males (OR: 0.73, 95% CI: 0.55–0.97, p = .03). Older youth were also less likely to be virally suppressed (OR: 0.68, 95% CI: 0.47–0.99, p = .05). as were participants who reported engaging in recent unprotected vaginal or anal sex (OR: .75, 95% CI: 0.58–0.98, p = .03). Youth with a high school education or greater (OR: 1.51, 95% CI: 1.13–2.02, p = .005) were more likely to be virally suppressed. Longer time since HIV diagnosis was also associated with likelihood of being virally suppressed (OR: 1.19, 95% CI: 1.14–1.25, p < .001). There were no significant associations between viral suppression and race, ethnicity, transgender identity, sexual orientation, employment status, mental health symptoms, or problematic substance use.

**Table 5:** Multi-level Associations between Viral Load Suppression and Structural- and Individual- Level Characteristics.

	Viral Load Suppression (Neighborhood Model)			Viral Load Suppression (Multilevel Model)		
	OR	95% CI	*p*	OR	95% CI	*p*
Mean Score, *β*_*0*_	0.37	(0.27,0.52)	.001	0.46	(0.28,0.76)	.003
Crime Index Score, *γ*_*01*_	0.99	(0.88,1.11)	.85	1.00	(0.88,1.13)	.99
Socioeconomic Disadvantage, *γ*_*02*_	0.89	(0.77,1.03)	.12	0.92	(0.78,1.09)	.35
% Black, *γ*_*03*_	1.00	(0.60,1.69)	.99	1.01	(0.56,1.82)	.97
% Hispanic, *γ*_*04*_	1.36	(0.74,2.51)	.33	1.22	(0.62,2.39)	.57
% Youth HIV Prevalence >13%, *γ*_*05*_	1.43	(1.17,1.75)	.001	1.50	(1.20,1.87)	.001
% Rurality > 5%, *γ*_*06*_	1.06	(0.64,1.76)	.82	0.93	(0.56,1.55)	.78
Behaviorally-infected, *β*_*1*_				1.25	(0.89,1.75)	.20
Cis-Female, *β*_*2*_				0.73	(0.55,0.97)	.03
Transgender, *β*_*3*_				0.61	(0.32,1.18)	.14
Aged 18 years of age or older, *β*_*4*_				0.68	(0.47,0.99)	.05
White Identified, *β*_*5*_				0.80	(0.60,1.07)	.14
Hispanic Ethnicity, *β*_*6*_				1.07	(0.76,1.51)	.71
Heterosexual identity, *β*_*7*_				0.99	(0.72,1.36)	.93
Employed, *β*_*8*_				1.18	(0.93,1.49)	.18
Completed High School, *β*_*9*_				1.51	(1.13,2.02)	.005
Mental Health Symptoms, *β*_*10*_				0.83	(0.65,1.06)	.13
Problematic Substance Use, *β*_*11*_				0.96	(0.73,1.26)	.78
Recent Unprotected Sex, *β*_*12*_				0.75	(0.58,0.98)	.03
Time Since Diagnosis, *β*_*13*_				1.19	(1.14,1.25)	.001

Notes. OR = Odds Ratio; CI = Confidence Interval; ART = Antiretroviral therapy

## Discussion

We sought to examine how multi-level structural and individual correlates were related to ART use (current and for at least 6 months), consistent HIV care appointment keeping, and viral suppression among behaviorally and perinatally HIV-infected adolescents and young adults in the United States. We highlight the complex relationship between these factors and HIV care outcomes below.

Adolescents and young adults living in more socioeconomically disadvantaged areas were less likely to report current ART use, even after accounting for individual level factors known to influence its use. This finding accords with other research that suggests that those living in areas with higher rates of unemployment are less likely to have a current ART prescription [23]. There are many complex issues related to availability of ART and utilization, including environmental and resource considerations. Our findings are consistent with other data reporting that proxies for poverty, including food insecurity, are associated with lacking access to ART and delaying engagement in HIV services both in the United States and internationally [38–40]. These findings suggest that structural factors may play an important role in understanding the reasons for failure in linkage to and engagement in care, and points to the importance of developing multi-level interventions focused on addressing the continuum of care. Specifically, efforts to strengthen linkage and retention in care may be warranted.

In contrast, however, after controlling for individual factors, we found that HIV-infected adolescents and young adults living in areas with greater economic disadvantage were more likely to report using ART consistently for at least 6 months. This is also somewhat consistent with findings indicating an inverse relationship between economic deprivation and residence in a location associated with poor retention in HIV care [25]. Furthermore, adolescents and young adults living in counties where more than 13% of all HIV cases were accounted for by 13–24 year olds were more likely to report current and ≥6 months of ART use as well as to be virally suppressed. Stated another way, adolescents and young adults who lived in counties where adolescents and young adults accounted for a larger proportion of the diagnosed HIV prevalence were more likely to be virally suppressed than counterparts living in areas with lower prevalence among youth. Programs across the United States (e.g., Ryan White HIV/AIDS Program/AIDS Drug Assistance Program, Medicaid) that provide services to individuals who do not have sufficient health care coverage or financial resources to cope with HIV should be targeted to areas in which HIV incidence, prevalence and burden are highest Thus, our findings may reflect the value of having safety net programs or effective outreach for HIV-infected individuals living in highly-impacted communities, particularly as it may highlight how having functional health care systems may help offset the deleterious health consequences associated with living in poverty.

Structural factors such as the availability of services are important but their influence may differ based on where youth are in the continuum of care. Our data point to the fact that the initiation of services (current ART use) may not be occurring in areas with greater socioeconomic disadvantage. Reasons for this are speculative at this point, but may include administrative burdens (both for youth and clinical settings) associated with initiating HIV services/entitlements (particularly for those who no longer are no longer covered by the parents’ Medicaid or lack motivated parents and significant others to monitor their loss of eligibility). Also, there are individual level factors that may make providers hesitant to start youth on ART (e.g., substance use, mental health; see Kahana et al. [4] for discussion on this) or stigma associated with asking providers for ART.

On the other hand, our data suggest that once youth start using ART, the likelihood that they engage and remain in HIV care (i.e., on ART ≥6 months) may increase and that this occurs in a variety of clinic settings (academic or other, as represented in the ATN). Though we did not collect insurance data for youth in this study, estimates from an ongoing cohort study within the ATN indicated that at least 40% of ATN youth had Medicaid, Medicare, or other publicly funded health coverage (D. Fortenberry, personal communication, April 2015). Conceptually, our findings are consistent with the theory of health care agglomeration on health outcomes [41, 42], which posits that locales that need a specific set of services (e.g., HIV care services) become more concentrated with organizations providing these services, and consequentially become more likely to increase engagement in continued care among their clientele even if these conditions do not necessarily foster the initial access and use of care. This suggests that the need for HIV care among youth and the presence of publicly funded entitlements for a large fraction of these youth may ultimately create sufficient infrastructure to support continued care and, ultimately, viral suppression on a geographically-defined population basis. Because testing and initial linkage to care often originate outside of HIV care settings, some of the geographic disadvantage gap in ART uptake may reflect systemic rather than geographic barriers [24].

The current findings highlight that racial disparities in continued ART use (as defined by ART use for at least 6 months) exist. At the individual-level, we observed that Whites were more likely than non-White counterparts to report continued ART use. Moreover, we observed a trend at the neighborhood-level (p = .06), whereby youth living in neighborhoods more densely populated by African Americans were less likely to report continued ART use. It is important to note that racial disparities for certain HIV outcomes, such as viral suppression rates, remain despite seemingly equal access to HIV care [43–46]. Distrust of health care among many African Americans is real and likely stems from a legacy of abuse, from medical experimentation on slaves to the unethical practices with patients in the Tuskegee Syphilis study. Experiences of racism, mistrust, conspiracy beliefs and the quality of provider relationships appeared to impact engagement in care, particularly for HIV care [47–49]. These norms among many in segregated, often isolated, African American communities may be exerting an effect on adolescents and young adults’ willingness to engage in continued HIV care (as defined by continued ART use) and thus result in racial health disparities [50–52]. Among HIV-uninfected African American MSM, those who experienced greater stigma from health care providers and global medical mistrust had longer gaps in time since their last medical exam [49].

Of course, there are also arguments to support that African Americans may have less ready access to HIV care, including infectious disease physicians as primary providers [50] and are more likely access HIV drugs through Medicaid than through ADAPs, which may reflect differences in program eligibility criteria as well as care seeking later in HIV disease [53]. African Americans have limited access to high-quality health care due to high rates of under- and uninsurance; nearly one fifth lacked health insurance in 2012 [54–56]. This limits access to HIV prevention and care, and may contribute to worse HIV health outcomes, including lower rates of linkage to care, retention in care, prescription for ART, and viral suppression.

Our findings point to the need for the implementation of structural-level interventions that can reduce disparities in health related outcomes. This is a recurring comment in the HIV prevention and care literature around the world [57]. Inadequate resources for meeting subsistence needs and gaining access to health care remain persistent barriers to adequate health among adult HIV populations [58] and our findings are likely to reflect similar resource inequities as they effect youth. Relatively few structural interventions have been done to promote HIV treatment and care, and the emphasis has been on individual-level interventions for clinic settings. The complexity of addressing multiple structural factors such as adequate housing and health care services makes it difficult to use clinical trial designs that typically shape policy and practice. Efforts to improve housing for people living with HIV, for example, have yielded mixed results, which were difficult to disentangle from activities outside of the actual intervention [59]. Interventions that address geographic variations in care and outcomes like the patterns evident here are particularly complex and spatial features of the environment may be confounded with racial and other structural factors. Attention to broader inequities, such as health care insurance coverage and access, particularly for minority youth, is critical to addressing the health disparities seen in our data.

The ATN has attempted to address structural factors in the public health and health care systems that affect entry into HIV care for youth [24]. This suggests a line of structural intervention research and practice that is proximal to the clinic environment but able to address some needs related to linking HIV-infected youth to care. The expansion of health care access under the Affordable Care Act is a broader structural intervention that potentially aids in the delivery of HIV care to youth, although capturing its effects may be complex. Improving access to services also may need to consider the lack of services directed at male youth (the majority of this sample). There are no specialty settings or disciplines that directly serve young males in the same way that reproductive health care reaches young females and efforts in this direction will need to consider how to identify geographic communities where this need is greatest and pair this with the outreach and social capital needed to actually engage young men. Finally, condom distribution programs are cost-effective structural interventions that provide communities with the resources they need to prevent HIV acquisition and secondary transmission [60].

The current findings must be interpreted in light of several limitations. The results are generalizable only to HIV-infected adolescents and young adults who have been diagnosed and linked to care and the cities and counties that were represented with the ATN sample. Future work should extend to current findings with the inclusion of more locales affected by HIV in the US. There are other structural factors that we did not measure which would certainly contribute to a richer dataset, such as travel distance and transportation to clinics and pharmacies to access care, both of which have been shown to be related to HIV care outcomes [22, 24]. We estimated insurance policy/eligibility (i.e., Ryan White) but did not actually collect these data. We used current ART use as a proxy of access to care or initiation of services and being on ART for at least 6 months as a proxy for continued care. Our missed HIV care appointment variable as a proxy for retention in care is arguably a somewhat crude measure. However, there is no gold standard for retention in care and our missed number of appointments variable has been associated with 12 month VL suppression rates [61]. Finally, some though not all of the individual level data used in the current analyses, such as current ART use, were self-reported. Future research would also be well-served to include participants’ perceptions of areas in which they reside, including such factors as social cohesion, attachment to and identification and involvement with neighborhood, and measurement of social norms relating to accessing health care, substance use and sexual behavior.

## Conclusions

We contribute to this body of literature by highlighting how structural-level factors, in concert with individual-level factors, are related to ART use, HIV care appointment attendance, and viral suppression for adolescents and young adults in the United States. We strongly encourage clinicians and researchers to consider these broader ecological factors as they implement interventions to improve targets aligned with the goals of the national HIV/AIDS strategy to decrease HIV incidence, improve health outcomes and reduce HIV-related health disparities. Furthermore, health care policies and legislation that acknowledge, incorporate, and address broader structural contexts and demographics (rather than a focus alone in individual risk factors) should be tied to resource allocation as they are more likely to ensure individuals’ positive health outcomes.

## Supporting Information

S1 Fig ATN nodes and surrounding areas from which youth in the current sample were recruited. (DOCX)
