# Supplementary material for: Structural Determinants of Antiretroviral Therapy Use, HIV Care Attendance, and Viral Suppression among Adolescents and Young Adults Living with HIV
Source: PLoS One. 2016 Apr 1;11(4):e0151106. doi: 10.1371/journal.pone.0151106 (PMC4817971; doi:10.1371/journal.pone.0151106)

Supporting Information

S1 Fig. ATN nodes and surrounding areas from which youth in the current sample were recruited


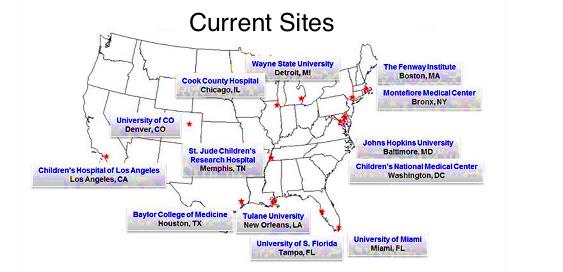

Supplement: S1 Fig — (DOCX) [file pone.0151106.s001.docx]
